# Skeletal effects of JAK1/2 inhibition versu*s* IL-6 receptor blockade in rheumatoid arthritis: a *post hoc* comparative cohort analysis based on two prospective observational studies

**DOI:** 10.3389/fphar.2026.1835307

**Published:** 2026-05-28

**Authors:** Nils Schulz, Pascal van Wijnen, Tim Wilhelmi, Ulf Müller-Ladner, Uwe Lange, Philipp Klemm

**Affiliations:** Department of Rheumatology, Justus Liebig University Giessen, Clinical Immunology, Osteology and Physical Medicine, Bad Nauheim, Germany

**Keywords:** baricitinib, bone mineral density, interleukin-6 receptor blockade, janus kinase inhibitor, osteoporosis, rheumatoid arthritis, tocilizumab

## Abstract

**Introduction:**

Janus kinase inhibitors (JAKi) are increasingly used in rheumatoid arthritis (RA), yet comparative data on systemic effects beyond joint inflammation remain limited. Because chronic inflammation promotes generalized bone loss and IL-6-driven osteoclastogenesis, we compared the skeletal effects of baricitinib and tocilizumab over 12 months.

**Methods:**

We performed an exploratory *post hoc* comparative cohort analysis based on two prospective single-center observational cohorts with harmonized eligibility criteria and identical endpoints. Patients with active RA initiating either baricitinib (n = 26) or tocilizumab (n = 22) underwent dual-energy X-ray absorptiometry (DXA) at baseline and after 12 months. Primary outcomes were changes in bone mineral density (BMD) at the lumbar spine and femoral neck. Secondary analyses included changes in disease activity (DAS28-CRP) and multivariable models to identify factors associated with BMD change.

**Results:**

No significant differences in BMD changes were observed between groups. Mean spine BMD increased by +8.2 mg/cm^2^ (+0.7%, 95% CI [−12.5.29.0]) with baricitinib and +15.6 mg/cm^2^ (+1.5%, 95% CI [−24.0.55.0]) with tocilizumab. Femoral BMD remained stable under baricitinib (+7.6 mg/cm^2^, +0.9%, 95% CI [−11.6.26.8]) but declined non-significantly with tocilizumab (−9.8 mg/cm^2^, -1.0%, 95% CI [−24.8.5.1]). Multivariable ANCOVA identified change in DAS28-CRP as the only factor significantly associated with spine BMD change (β = −0.36; p = 0.027). Treatment type, glucocorticoid exposure, and autoantibody status were not independently associated with BMD outcomes.

**Conclusion:**

JAK1/2 inhibition with baricitinib and IL-6 receptor blockade with tocilizumab showed comparable 12-month effects on lumbar spine and femoral neck BMD. These findings suggest that preservation of bone health in RA is more closely linked to effective suppression of inflammatory activity than to a detectable advantage of one targeted mechanism over the other.

## Introduction

Patients with rheumatoid arthritis (RA) exhibit a substantially increased risk of osteoporosis compared with the general population (Moshayedi et al., 2022). Chronic systemic inflammation in RA leads to elevated concentrations of proinflammatory cytokines such as tumor necrosis factor alpha (TNFα), interleukin (IL)-1β and IL-6. These mediators drive osteoclast differentiation and activity while simultaneously suppressing osteoblast function resulting in enhanced bone resorption and impaired bone formation (Pietschmann et al., 2022; Zhou et al., 2022). In addition anti-citrullinated protein antibodies (ACPA) emerge early in the disease course and directly promote osteoclastogenesis thereby contributing to systemic bone loss even before the onset of clinical symptoms (Kocijan et al., 2013). Pain related immobility, progressive muscle weakness and functional impairment further accelerate loss of bone mineral density (BMD) and increase the likelihood of secondary osteoporosis (Baker et al., 2022). In addition to chronic inflammation and autoantibody-mediated mechanisms, glucocorticoids represent an important contributor to secondary osteoporosis in RA, particularly in the presence of uncontrolled inflammation (Wiebe et al., 2022).

Disease modifying antirheumatic drugs (DMARDs) remain the cornerstone of RA management and encompass conventional synthetic (cs), biologic (b) and targeted synthetic (ts) DMARDs. Over the past years, it has become increasingly evident that DMARDs can exert bone-protective effects beyond their anti-inflammatory action (Hauser et al., 2022). Importantly, there are also clear differences in the osteoprotective effects among individual DMARDs, even when comparable improvements in disease activity are achieved. While csDMARDs such as methotrexate (MTX) or the bDMARD rituximab have been shown to reduce joint inflammation effectively, they fail to confer measurable benefit on BMD (Buckley et al., 1997; Wheater et al., 2018). In contrast, TNFα inhibitors showed an osteoprotective effect even independent of the RA treatment response (Al-Bogami et al., 2021). These findings suggest additional mechanisms of action, such as direct effects on cells and bone homeostasis, that may vary significantly between different drugs.

Emerging evidence from a retrospective cohort of 362 patients with RA implies that janus kinase inhibitors (JAKi, tsDMARDs) may exert superior osteoprotective effects relative to both cs and bDMARDs (Chen et al., 2024). Mechanistically, JAKi modulate JAK/signal transducer and activator of transcription (STAT) signaling pathways that are central to the regulation of the receptor activator of nuclear factor kappa B ligand (RANKL)/osteoprotegrin (OPG) system thereby inhibiting osteoclastogenesis and enhancing osteoblast function (Adam et al., 2020; Hu et al., 2022). Unlike most bDMARDs, which block individual cytokines, JAKi act on a broad spectrum of proinflammatory mediators and exhibit senomorphic properties, attenuating the senescence-associated secretory phenotype (SASP) and thereby potentially further improving bone remodeling (Farr et al., 2017; Xu et al., 2015).

Taken together, these observations suggest that targeted therapies in RA may differ not only in their anti-inflammatory efficacy but also in their effects on systemic bone health. In this context, JAKi are of particular interest because their broader modulation of cytokine signaling may influence skeletal outcomes beyond joint inflammation. However, comparative clinical data on the skeletal effects of JAK inhibition and IL-6 blockade are lacking. We therefore aimed to compare the 12-month effects of baricitinib and tocilizumab on BMD in patients with RA. Secondary objectives included the comparison of changes in disease activity (Δ Disease Activity Score 28-C-reactive protein, DAS28-CRP) and glucocorticoid dose over 12 months, as well as the identification of factors independently associated with BMD change using multivariable models.

## Materials and methods

### Study design and setting

We performed a *post hoc* comparative cohort analysis based on two previously conducted single-center prospective observational cohort studies at our center. Both studies were carried out at the Department of Rheumatology, Clinical Immunology, Osteology, and Physical Medicine of the Justus Liebig University Giessen, Campus Kerckhoff, a tertiary academic center in Germany. The cohorts followed comparable prospective research protocols, with similar inclusion and exclusion criteria, endpoints and assessment methods. Although randomization was not performed, the analytic design aligned eligibility criteria, observation periods, and outcome definitions to approximate the structure of a randomized comparative analysis. The baricitinib cohort (Bone mineral density during treatment with the Janus kinase inhibitor baricitinib in patients with rheumatoid arthritis: a monocentric observational study (Schu et al., 2025)) was recruited between 2019 and 2021; the tocilizumab cohort (Effects of Tocilizumab Therapy on Bone Mineral Density, Markers of Bone Metabolism and Disease Activity in TNF-α Inadequate Responders with Active Rheumatoid Arthritis (Wettich et al., 2014)) between 2010 and 2013.

### Participants

Eligibility criteria were harmonized across both studies. Included patients had to:

- meet the 2010 ACR/EULAR classification criteria for RA (Kay and Upchurch, 2012),
- have active RA (DAS28-CRP >3.2),
- start treatment with either baricitinib (4 mg/day, orally) or intravenous tocilizumab (8 mg/kg monthly),
- undergo dual-energy X-ray absorptiometry (DXA)-based BMD measurement of the lumbar spine (L1–L4) and femoral neck at baseline (treatment initiation) and after 12 months,
- have complete documentation of disease activity, glucocorticoid therapy, and autoantibody status including rheumatoid factor (RF) and ACPA.

Patients with other metabolic bone disorders, malignancies, or incomplete follow-up data were excluded. Concomitant osteoporosis medications (bisphosphonates, denosumab, teriparatide) were recorded at baseline and follow-up in both cohorts.

### Interventions

Baricitinib was administered orally at a daily dose of 4 mg. Tocilizumab was administered intravenously at a dose of 8 mg/kg every 4 weeks. Treatment regimens followed current German and European Alliance of Associations for Rheumatology (EULAR) guidelines at that time and were initiated as part of routine care. Concomitant therapy with csDMARDs and glucocorticoids was allowed and monitored. Changes in prednisolone dosage were recorded at baseline and at 12 months.

### BMD assessment

BMD was measured by DXA using a Lunar Prodigy X device (Lunar Radiation Corporation, Madison, Wisconsin, United States) in both studies. Measurements were performed at the lumbar spine (L1–L4) and the femoral neck. BMD values were recorded in g/cm^2^. All measurements were conducted in a standardized setting by trained technicians using consistent software and positioning protocols.

### Assessment of disease activity

Disease activity was assessed using the DAS28-CRP, a validated composite index based on the tender joint count (TJC28), swollen joint count (SJC28), patient global assessment of disease activity (PGA, visual analogue scale, 0–100 mm), and serum CRP levels (mg/dL) (Anderson et al., 2011). DAS28-CRP values were calculated at baseline and after 12 months using the formula recommended by the EULAR. Higher scores reflect greater disease activity. A score below 2.6 indicates remission.

### Outcomes

The primary endpoint was the absolute change in BMD (mg/cm^2^) at the lumbar spine and femoral neck over 12 months. Secondary endpoints included:

- relative (%) change in BMD at both sites,
- change in disease activity (ΔDAS28-CRP),
- change in daily prednisolone dose,
- identification of factors independently associated with BMD change using multivariable models.

Adverse events were recorded in the original studies. However, safety outcomes were not part of this analysis, as the present study focused exclusively on bone-related outcomes.

### Sample size

All patients fulfilling eligibility criteria and completing the 12-month follow-up were included in the analysis (n = 48: 26 baricitinib, 22 tocilizumab). Since no formal sample size calculation was performed in advance, this *post hoc* comparative cohort analysis should be considered exploratory.

### Statistical analysis

Descriptive statistics were presented as mean ± SD or median [IQR] for continuous variables, and frequencies (%) for categorical data. Between-group differences in baseline characteristics were assessed using Welch’s t-tests for continuous variables, Mann-Whitney U tests for skewed variables (disease duration, prednisolone doses, C-reactive protein, CRP), and Fisher’s exact tests for categorical variables. Within-group changes in BMD from baseline to 12 months were analyzed using paired t-tests. For the primary between-group comparisons of BMD change, unadjusted analyses were performed using Welch’s t-tests on absolute changes (ΔBMD). To account for potential confounding and baseline differences, analysis of covariance (ANCOVA) models were additionally constructed with the 12-month BMD value as the dependent variable, baseline BMD as a covariate, and treatment group (baricitinib vs. tocilizumab) as a fixed factor. Extended multivariable ANCOVA models further included age, sex, disease duration, autoantibody status (RF, ACPA), change in DAS28-CRP, and change in daily prednisolone dose as covariates. Effect estimates were reported as standardized β-coefficients with 95% confidence intervals (CIs). Model assumptions (normal distribution of residuals and homoscedasticity) were assessed visually using QQ-plots. Variance inflation factors (VIF) were calculated for all predictors to assess multicollinearity.

To assess the robustness of the primary findings with respect to the observed baseline imbalance in concomitant MTX use, an additional sensitivity analysis was performed in which baseline MTX use was added as a binary covariate to the extended multivariable models.

Post-hoc power analyses were performed to estimate the minimum detectable between-group effect size at 80% power and α = 0.05 (two-sided). To illustrate the association between change in disease activity (ΔDAS28-CRP) and spine BMD change, simple linear regression models were calculated (Figure 3).

All statistical analyses were conducted using R version 4.4.1 for Windows. A p-value <0.05 was considered statistically significant. All analyses were based on complete case data. No imputation for missing values was applied.

### Ethical considerations

Both studies received approval from the institutional ethics committee (baricitinib: reference no. AZ 149/18; tocilizumab: reference no. AZ 164/09).

## Results

### Patient characteristics

A total of 48 patients with RA were included in the analysis, of whom 26 received baricitinib and 22 received tocilizumab. The two groups were comparable in terms of age (baricitinib: 62.0 ± 10.5 years; tocilizumab: 62.6 ± 11.2 years) and disease duration (8.0 [5.0–13.5] vs. 9.5 [5.2–12.0] years, respectively).

Baseline and 12-month disease activity, assessed by DAS28-CRP, did not differ significantly between treatment groups, with scores declining from 4.35 ± 0.78 to 3.59 ± 1.12 in the baricitinib group and from 4.92 ± 1.45 to 3.50 ± 1.46 in the tocilizumab group (baseline: p = 0.110; 12 months: p = 0.804). The mean change in DAS28-CRP over 12 months was −0.76 ± 1.20 in the baricitinib group and −1.43 ± 1.34 in the tocilizumab group (p = 0.080).

However, patients in the tocilizumab group were significantly more often positive for ACPA and RF: ACPA positivity was present in 82% vs. 42% (p = 0.008), and RF positivity in 77% vs. 42% (p = 0.020). Initial prednisolone doses were also significantly higher in the tocilizumab group (5.0 [0.0–11.9] vs. 12.5 [10.0–20.0] mg/day, p < 0.001), while the mean daily dose at 12 months was comparable (0.0 [0.0–4.8] vs. 2.0 [1.0–3.0] mg/day, p = 0.276). Notably, 15 of 26 patients (57.7%) treated with baricitinib were prednisolone-free at 12 months, compared to five of 22 patients (22.7%) in the tocilizumab group (p = 0.020). This difference may also reflect the fact that fewer patients in the baricitinib group received glucocorticoids at treatment initiation (58% vs. 100%) and that the starting dose was substantially higher in the tocilizumab cohort.

Osteoporosis medication was used by 6 of 26 patients (23%) in the baricitinib group, whereas none of the tocilizumab-treated patients received osteoporosis therapy.

A complete overview of patient characteristics is provided in Table 1.

**TABLE 1:** Patient characteristics of the different RA cohorts.

Characteristic	Baricitinib (n = 26)	Tocilizumab (n = 22)	p-value
Female	20 (77%)	16 (73%)	0.751
Age (years)	62.0 ± 10.5	62.6 ± 11.2	0.832
Disease duration (years)	8.0 [5.0–13.5]	9.5 [5.2–12.0]	0.868
RF positive	11 (42%)	17 (77%)	0.020
ACPA positive	11 (42%)	18 (82%)	0.008
Previous b/tsDMARDs	1.8 ± 1.7	1.2 ± 0.4	0.245
Patients receiving prednisolone baseline	15 (58%)	22 (100%)	<0.001
Patients receiving prednisolone endpoint	11 (42%)	17 (77%)	0.020
Daily prednisolone dose (mg/day) baseline	5.0 [0.0–11.9]	12.5 [10.0–20.0]	<0.001
Daily prednisolone dose (mg/day) endpoint	0.0 [0.0–4.8]	2.0 [1.0–3.0]	0.276
Concomitant methotrexate therapy	8 (31%)	19 (86%)	<0.001
Osteoporosis therapy	6 (23%)	0 (0%)	0.025
DXA parameters			
BMD spine (g/cm^2^) baseline	1.12 ± 0.17	1.04 ± 0.21	0.189
BMD spine (g/cm^2^) endpoint	1.13 ± 0.18	1.06 ± 0.21	0.230
T-score spine baseline	−0.58 ± 1.40	−1.23 ± 1.85	0.203
T-score spine endpoint	−0.50 ± 1.46	−1.10 ± 1.80	0.243
BMD femoral neck (g/cm^2^) baseline	0.85 ± 0.14	0.91 ± 0.20	0.266
BMD femoral neck (g/cm^2^) endpoint	0.85 ± 0.15	0.90 ± 0.20	0.433
T-score femoral neck baseline	−1.33 ± 1.17	−1.00 ± 1.49	0.412
T-score femoral neck endpoint	−1.29 ± 1.20	−1.06 ± 1.46	0.568
RA disease activity			
CRP (mg/dL) baseline	0.50 [0.15–0.88]	0.95 [0.40–2.47]	0.099
CRP (mg/dL) endpoint	0.10 [0.10–0.78]	0.15 [0.10–0.62]	0.701
DAS28-CRP baseline	4.35 ± 0.78	4.92 ± 1.45	0.110
DAS28-CRP endpoint	3.59 ± 1.12	3.50 ± 1.46	0.804

Normally distributed continuous variables are presented as mean ± standard deviation (SD); non-normally distributed continuous variables (disease duration, prednisolone doses, CRP) are presented as median [interquartile range, IQR]. Categorical variables are presented as n (%).

Abbreviations: ACPA, anticitrullinated protein antibody; BMD, bone mineral density; b/tsDMARD, biological/target synthetic disease-modifying anti-rheumatic drug; CRP, C-reactive protein; DAS28, Disease Activity Score 28; DXA, dual-energy X-ray absorptiometry; n, number; RF, rheumatoid factor.

### Change in BMD

#### Spine BMD

After 12 months, mean lumbar spine BMD increased slightly in both groups (baricitinib: +8.2 mg/cm^2^ 95% CI [–12.5.29.0], corresponding to +0.7%; tocilizumab: +15.6 mg/cm^2^, 95% CI [–24.0.55.0], corresponding to +1.5%). Changes over time within each group were not statistically significant (paired t-test: p = 0.444 and p = 0.450, respectively).

**FIGURE 1:**
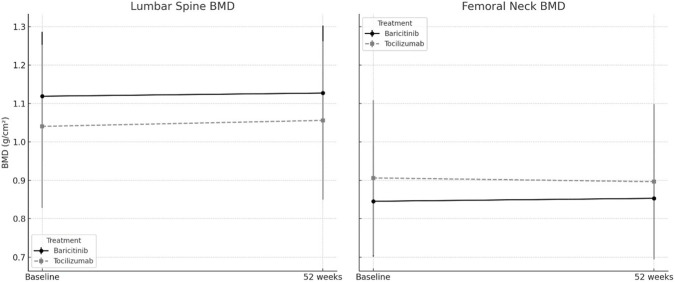
Mean bone mineral density (BMD) at baseline and after 52 weeks in patients with rheumatoid arthritis treated with baricitinib or tocilizumab. Left: lumbar spine (L1–L4); right: femoral neck. Values are presented as mean ± standard deviation (g/cm^2^). No statistically significant changes were observed within or between treatment groups.

The between-group difference in change was −7.4 mg/cm^2^ (95% CI [–54.0.39.0]; Welch’s t-test p = 0.748). In ANCOVA adjusted for baseline spine BMD, the treatment effect was not significant (p = 0.932) (*cf.* Figure 1).

#### Femoral neck BMD

Femoral neck BMD remained stable under baricitinib (+7.6 mg/cm^2^, 95% CI [–11.6.26.8], equivalent to +0.9%) and showed a non-significant decrease under tocilizumab (−9.8 mg/cm^2^, 95% CI [–24.8.5.1], equivalent to −1.0%). Changes over time within each group were not statistically significant (paired t-test: p = 0.445 and p = 0.213, respectively).

The between-group difference in change was +17.4 mg/cm^2^ (95% CI [–7.6.42.5]; Welch’s t-test p = 0.168). ANCOVA adjusted for baseline femoral BMD also revealed no significant group effect (p = 0.209) (*cf.* Figure 1).

### Impact of osteoporosis therapy within the baricitinib cohort

In the baricitinib cohort, 6 of 26 patients (23%) received osteoporosis medication (alendronate n = 3, zoledronate n = 1, ibandronate n = 1, denosumab n = 1), whereas none of the patients treated with tocilizumab were on anti-osteoporotic therapy. To assess whether this imbalance could have influenced our findings, we performed within-group analyses in the baricitinib arm. For the lumbar spine, the 12-month change in BMD did not differ significantly between patients with and without osteoporosis therapy (Welch’s t-test p = 0.726). In an ANCOVA model with 12-month lumbar BMD as the dependent variable, baseline BMD as covariate and osteoporosis therapy as a fixed factor, osteoporosis treatment was not independently associated with lumbar BMD at follow-up (β = 0.030; p = 0.672). A similar pattern was observed at the femoral neck: changes in femoral BMD were not significantly different between patients with and without osteoporosis medication (Welch’s t-test p = 0.470), and in the corresponding ANCOVA model osteoporosis therapy did not emerge as an independent predictor of 12-month femoral BMD (β = −0.054; p = 0.483). Baseline BMD did not differ relevantly between patients with and without osteoporosis medication, and treated individuals did not display outlier trajectories.

### Associations with BMD change

**FIGURE 2:**
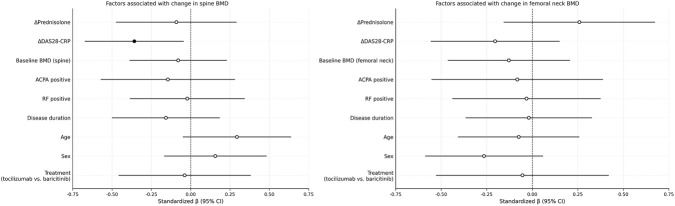
Associations with change in bone mineral density (BMD) at the spine and femoral neck over 12 months. Forest plots illustrate standardized β-coefficients with 95% confidence intervals (CI) from multivariable analysis of covariance (ANCOVA) models assessing factors associated with absolute change in lumbar spine (left) and femoral neck (right) BMD. Variables included in the analysis included treatment (tocilizumab vs. baricitinib), sex, age, disease duration, autoantibody status [rheumatoid factor (RF) and/or anti-citrullinated protein antibodies (ACPA)], baseline BMD at the respective site, change in disease activity [change in Disease Activity Score 28 - C-reactive protein (ΔDAS28-CRP)], and change in daily prednisolone dose. Only reduction in disease activity was significantly associated with spine BMD change (standardized β = −0.36; 95% CI [–0.67,–0.04]; p = 0.027). No other covariates were significantly associated with changes in BMD at either site.

In the multivariable ANCOVA model for lumbar spine BMD, reduction in disease activity (ΔDAS28-CRP) was the only independent factor significantly associated with spinal bone preservation (standardized β = −0.36; 95% CI [−0.67,−0.04]; p = 0.027). Of note, none of the other covariates were significant (all p > 0.09, *cf.* Figure 2). The overall model fit was not statistically significant (p = 0.11).

In contrast, the femoral neck model did not reveal any significant factor associated with BMD change. Neither ΔDAS28 (p = 0.25) nor any of the clinical or serological variables were independently associated with femoral BMD (*cf.* Figure 2). The overall model was not significant (p = 0.384).

**FIGURE 3:**
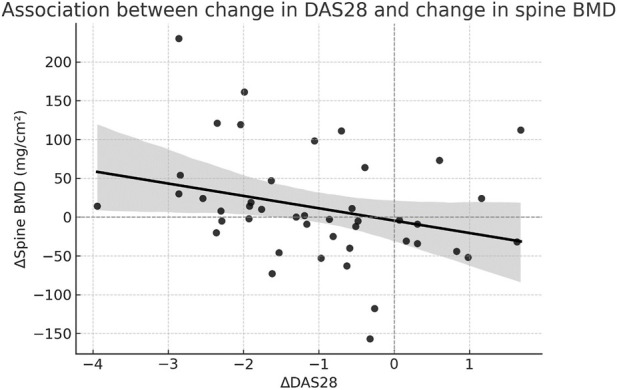
Association between change in disease activity [change in Disease Activity Score 28 - C-reactive protein (ΔDAS28-CRP)] and change in lumbar spine bone mineral density (Δspine BMD) over 12 months. In a simple linear regression, no significance could be observed (standardized β = −0.29; 95% CI [–0.64.0.02]; p = 0.057).

In a simple linear regression, ΔDAS28 was not significantly associated with spine BMD change (standardized β = −0.29; 95% CI [–0.64.0.02]; p = 0.057; *cf.* Figure 3).

### Sensitivity analyses including MTX use

Because baseline MTX use differed substantially between groups, additional sensitivity analyses were performed including MTX use as a binary covariate. In the lumbar spine model, treatment group remained not significantly associated with BMD change, and MTX use was not independently associated with the outcome (standardized β = 0.09; 95% CI [–0.29.0.46]; p = 0.634). The association between ΔDAS28-CRP and lumbar spine BMD change remained significant after inclusion of MTX use (standardized β = −0.38; 95% CI [–0.71,–0.05]; p = 0.026). In the femoral neck model, neither MTX use (standardized β = −0.04; p = 0.823) nor treatment group was significantly associated with BMD change. VIF analysis did not indicate relevant multicollinearity, with all VIF values remaining below 3.5 in the MTX-adjusted models (*cf.* Supplementary Table S1).

### Post-hoc power analysis

Post-hoc estimation of the minimum detectable effect size showed that, with the observed sample sizes, the study had 80% power only to detect large between-group differences corresponding to Cohen’s d ≥ 0.830, equivalent to approximately 59.6 mg/cm^2^ at the lumbar spine and 35.7 mg/cm^2^ at the femoral neck.

## Discussion

In this *post hoc* comparative cohort analysis, combining data from two independent prospective observational cohort studies, we compared the 12-month skeletal effects of JAK1/2 inhibition with baricitinib and IL-6 receptor blockade with tocilizumab in patients with RA. To date, no prospective studies have compared different DMARDs with respect to bone-specific outcomes (Hauser et al., 2022). This analysis therefore addresses an important evidence gap in comparative evaluation of targeted therapies with regard to skeletal health, particularly as both studies prospectively assessed BMD as a predefined primary outcome.

In the direct comparison, no statistically significant difference was observed between the treatment groups regarding changes in lumbar spine or femoral neck BMD. Consistent with findings from previous studies, BMD changes within the individual groups were also not significant (Schu et al., 2025; Wettich et al., 2014). Notably, mean spine BMD remained stable in both groups (baricitinib +0.73%, tocilizumab +1.5%), while a non-significant reduction of approximately 1% in femoral neck BMD was observed in the tocilizumab group (baricitinib +0.90%). Changes of a similar magnitude have been observed in other studies investigating tocilizumab, although those reported a numerical increase in femoral BMD (Chen et al., 2017; Kume et al., 2014). To date, no comparable study has been conducted for baricitinib. The observed changes in BMD appear clinically relevant when considering that healthy individuals typically experience annual BMD losses of 0.2% at the spine and 0.3%–0.5% at the femur (Cvijetić and Koršić, 2004), while the annual BMD loss in RA patients is even more pronounced (spine: −1.75%, femur: −1.4%) (Huang et al., 2022). The overall stability of spine BMD and the modest changes at the femoral site observed in our study therefore may reflect a clinically meaningful preservation of bone health under effective immunomodulatory therapy.

Although baricitinib and tocilizumab differ in their molecular targets and mechanisms of action, the absence of a detectable difference in BMD outcomes may reflect comparable effects on key inflammatory pathways relevant to bone loss. Both drugs modulate IL-6 signalling, which represents a pivotal cytokine in inflammation-driven osteoclastogenesis in RA by upregulating RANKL expression on osteoblasts and synovial fibroblasts (Hu et al., 2022; Murakami et al., 2017; Schett et al., 2021). Tocilizumab achieves this through direct blockade of the IL-6 receptor, whereas baricitinib exerts its effects by inhibiting JAK1 and JAK2, thereby attenuating downstream signalling of IL-6 along with additional pro-inflammatory cytokines including GM-CSF and interferon-dependent pathways (Hu et al., 2022; Schett et al., 2021). While the broader immunomodulatory profile of baricitinib might suggest a theoretical advantage, it is plausible that the shared suppression of IL-6-mediated osteoclast activation constitutes the key mechanism underpinning bone preservation. In the context of well-controlled inflammation, as was the case in both cohorts, additional cytokine inhibition did not result in an additional osteoprotective benefit within a 12-month timeframe. This pathophysiological overlap may help explain the comparable clinical outcomes observed in this study.

Our findings differ from those of a recently published retrospective cohort study that reported greater increases in BMD under JAKi therapy compared to non-TNF biologics (Chen et al., 2024). However, that analysis had several methodological limitations: (i) significant group size imbalance (JAKi n = 30 vs. non-TNF bDMARDs n = 108) without differentiation among individual biologics, (ii) substantially shorter interval between DXA assessments in the JAKi group (median 1.1 vs. 2.8–5.0 years in other groups), (iii) imbalances in baseline characteristics including higher rates of antiresorptive therapy and lower osteoporosis prevalence in the JAKi group, and (iv) lack of adjustment for change in disease activity - a key driver of bone loss, as shown in the literature and underscored by our multivariable analysis (Buttgereit et al., 2024). Taken together, these differences underscore the need for adequately controlled, drug-specific analyses when evaluating bone-related outcomes under DMARD therapy.

Multivariable ANCOVA models confirmed that change in disease activity, measured by DAS28, was the only independent factor significantly associated with spine BMD change (β = −0.36; 95% CI [−0.67,−0.04]; p = 0.027). Greater reductions in disease activity were associated with less spine bone loss. In contrast, in the model for femoral neck BMD no individual factor emerged as significant. Neither the treatment type, glucocorticoid exposure, nor autoantibody status had a significant independent effect on BMD outcomes in the adjusted models. These findings suggest that the initially observed differences between treatment groups, particularly regarding baseline glucocorticoid use and autoantibody status, may have had limited impact on bone outcomes.

These findings are consistent with previous observations suggesting that low-dose glucocorticoid therapy (≤5 mg/day) may not exert a detrimental effect on bone when inflammation is adequately suppressed, whereas higher doses (>7.5 mg/day) appear to pose a risk primarily in the setting of uncontrolled disease activity (Wiebe et al., 2022). Similarly, although ACPA has been implicated in promoting systemic bone loss through enhanced osteoclastogenesis (Kocijan et al., 2013), no significant association with BMD change was observed in this cohort - likely reflecting the overriding influence of effective inflammation control. Taken together, these results support the concept that bone preservation in RA is more closely related to the degree of inflammatory control than to a detectable advantage of one targeted mechanism over another within the present observation period.

The imbalance in osteoporosis medication use (23% in the baricitinib cohort vs. 0% in the tocilizumab cohort) represents a relevant difference between groups. However, within-cohort analyses in the baricitinib arm showed no significant effect of osteoporosis therapy on lumbar or femoral BMD trajectories, neither in unadjusted comparisons nor in baseline-adjusted models, and treated individuals did not display outlier patterns. Together with comparable baseline BMD between patients with and without osteoporosis medication, these findings suggest that anti-osteoporotic treatment had only limited influence on the observed BMD changes, although residual confounding cannot be fully excluded. Moreover, concomitant MTX use and baseline prednisolone dose were not included in the primary multivariable ANCOVA model–the former based on established evidence that MTX does not independently affect BMD (Buckley et al., 1997), and the latter because change in prednisolone dose was already included as a covariate, capturing the therapeutic glucocorticoid reduction over the observation period. Nevertheless, the substantial baseline imbalances in these variables (MTX use: 31% vs. 86%, baseline prednisolone: 5.0 [0.0–11.9] vs. 12.5 [10.0–20.0] mg/day) represent relevant potential confounders. Sensitivity analyses incorporating MTX as an additional binary covariate confirmed the robustness of the primary findings: MTX was not significantly associated with BMD outcomes at either skeletal site (spine: β = +0.088, p = 0.634; femoral neck: β = −0.043, p = 0.823), and ΔDAS28-CRP remained the only significant factor associated with spine BMD change across all adjusted models (p range: 0.026–0.027).

A further limitation is the 12-month interval between DXA assessments, which may be insufficient to capture the full trajectory of treatment-related bone remodeling. Notably, however, the identical observation period across both groups avoids the between-group disparities in follow-up duration that complicate the interpretation of retrospective analyses such as Chen et al. (Chen et al., 2024), as discussed above. Additional limitations include the relatively small sample size and the complete-case approach without an *a priori* sample size calculation, both of which reduce statistical power and increase the risk of type II error. Post-hoc power analysis indicated that the study had 80% power only to detect large between-group effects of Cohen’s d ≥ 0.830, corresponding to BMD differences of approximately 59.6 mg/cm^2^ at the lumbar spine and 35.7 mg/cm^2^ at the femoral neck. Therefore, the absence of statistically significant between-group differences should be interpreted with caution as subtle or moderate mechanism-specific differences may have remained undetected.

Treatment allocation was not randomized, and residual confounding remains possible despite adjustment for key covariates, including glucocorticoid exposure and autoantibody status. The two cohorts were recruited in different calendar periods, with the tocilizumab cohort enrolled between 2010 and 2013 and the baricitinib cohort between 2019 and 2021. During this interval, RA management evolved substantially, including broader implementation of treat-to-target strategies, more structured disease activity monitoring, changes in glucocorticoid tapering, and increasing availability of targeted therapies (Smolen et al., 2010). These secular changes may have influenced treatment patterns, concomitant MTX use, glucocorticoid exposure, and osteoporosis management. This temporal confounding represents an inherent limitation of combining historical and contemporary cohort data.

Furthermore, harmonized comorbidity data were not available for both cohorts. The tocilizumab cohort (Wettich et al., 2014) did not include systematic comorbidity documentation beyond the exclusion of conditions known to affect bone metabolism. Consequently, a meaningful between-group comparison of comorbidity burden could not be performed and residual confounding from unrecorded comorbidities cannot be fully excluded.

Finally, DXA-based BMD measurements do not capture alterations in bone microarchitecture or quality (Boyadzhieva et al., 2023). Bone turnover markers such as serum cross-linked C-telopeptide of type I collagen (CTX) or procollagen type I N-terminal propeptide (P1NP) were not available from both cohorts in a harmonized format and could not be included in the present analysis. Their availability could have provided important mechanistic insight into the kinetics of bone resorption and formation under each treatment, thereby strengthening the biological plausibility of the observed BMD findings (Vasikaran et al., 2011). Future studies should aim to include head-to-head comparisons of drugs with distinct cytokine-targeting profiles, such as TNFα inhibitors versus JAKi, to further elucidate class-specific effects on bone outcomes (Zhou et al., 2022; Hauser et al., 2022).

## Conclusion

In conclusion, JAK1/2 inhibition with baricitinib and IL-6 receptor blockade with tocilizumab showed comparable 12-month effects on lumbar spine and femoral neck BMD. These findings suggest that preservation of bone health in RA is more closely linked to effective suppression of inflammatory activity than to a detectable advantage of one targeted mechanism over the other.

## Data Availability

The data that support the findings of this study are available from the corresponding author upon reasonable request.

## References

[B1] Adam S. Simon N. Steffen U. Andes F. T. Scholtysek C. Müller D. I. H. (2020). JAK inhibition increases bone mass in steady-state conditions and ameliorates pathological bone loss by stimulating osteoblast function. Sci. Transl. Med. 12, eaay4447. 10.1126/scitranslmed.aay4447 32051226

[B2] Al-Bogami M. Bystrom J. Clanchy F. Taher T. E. Mangat P. Williams R. O. (2021). TNFα inhibitors reduce bone loss in rheumatoid arthritis independent of clinical response by reducing osteoclast precursors and IL-20. Rheumatol. Oxf. 60, 947–957. 10.1093/rheumatology/keaa551 32984900

[B3] Anderson J. K. Zimmerman L. Caplan L. Michaud K. (2011). Measures of rheumatoid arthritis disease activity: patient (PtGA) and provider (PrGA) global assessment of disease activity, disease activity score (DAS) and disease activity score with 28-Joint counts (DAS28), simplified disease activity index (SDAI), clinical disease activity index (CDAI), patient activity score (PAS) and patient activity Score-II (PASII), routine assessment of patient index data (RAPID), rheumatoid arthritis disease activity index (RADAI) and rheumatoid arthritis Disease activity Index-5 (RADAI-5), chronic arthritis systemic index (CASI), patient-based disease activity score with ESR (PDAS1) and patient-based disease activity score without ESR (PDAS2), and mean overall index for rheumatoid arthritis (MOI-RA). Arthritis Care Res. Hob. 63 (11), S14–S36. 10.1002/acr.20621 22588741

[B4] Baker R. Narla R. Baker J. F. Wysham K. D. (2022). Risk factors for osteoporosis and fractures in rheumatoid arthritis. Best. Pract. Res. Clin. Rheumatol. 36, 101773. 10.1016/j.berh.2022.101773 36208961

[B5] Boyadzhieva Z. Palmowski A. Buttgereit F. Hoff P. (2023). Trabecular bone score in rheumatology: are there benefits in comparison to bone densitometry alone? Z Rheumatol. 82, 672–677. 10.1007/s00393-023-01407-5 37646845

[B6] Buckley L. M. Leib E. S. Cartularo K. S. Vacek P. M. Cooper S. M. (1997). Effects of low dose methotrexate on the bone mineral density of patients with rheumatoid arthritis. J. Rheumatol. 24, 1489–1494. 9263140

[B7] Buttgereit F. Palmowski A. Bond M. Adami G. Dejaco C. (2024). Osteoporosis and fracture risk are multifactorial in patients with inflammatory rheumatic diseases. Nat. Rev. Rheumatol. 20, 417–431. 10.1038/s41584-024-01120-w 38831028

[B8] Chen Y.-M. Chen H.-H. Huang W.-N. Liao T. L. Chen J. P. Chao W. C. (2017). Tocilizumab potentially prevents bone loss in patients with anticitrullinated protein antibody-positive rheumatoid arthritis. PLoS One 12, e0188454. 10.1371/journal.pone.0188454 29155868 PMC5695761

[B9] Chen Y.-W. Chen H.-H. Huang W.-N. Chen J. P. Chen Y. M. (2024). Potential alleviation of bone mineral density loss with janus kinase inhibitors in rheumatoid arthritis. Clin. Rheumatol. 43, 117–128. 10.1007/s10067-023-06735-0 37658935

[B10] Cvijetić S. Koršić M. (2004). Apparent bone mineral density estimated from DXA in healthy men and women. Osteoporos. Int. 15, 295–300. 10.1007/s00198-003-1525-x 14628108

[B11] Farr J. N. Xu M. Weivoda M. M. Monroe D. G. Fraser D. G. Onken J. L. (2017). Targeting cellular senescence prevents age-related bone loss in mice. Nat. Med. 23, 1072–1079. 10.1038/nm.4385 28825716 PMC5657592

[B12] Hauser B. Raterman H. Ralston S. H. Lems W. F. (2022). The effect of anti-rheumatic drugs on the skeleton. Calcif. Tissue Int. 111, 445–456. 10.1007/s00223-022-01001-y 35771255 PMC9560949

[B13] Hu L. Liu R. Zhang L. (2022). Advance in bone destruction participated by JAK/STAT in rheumatoid arthritis and therapeutic effect of JAK/STAT inhibitors. Int. Immunopharmacol. 111, 109095. 10.1016/j.intimp.2022.109095 35926270

[B14] Huang H. Wang Y. Xie W. Geng Y. Gao D. Zhang Z. (2022). Impact of treat-to-target therapy on bone mineral density loss in patients with rheumatoid arthritis: a prospective cohort study. Front. Endocrinol. (Lausanne) 13, 867610. 10.3389/fendo.2022.867610 35655798 PMC9152020

[B15] Kay J. Upchurch K. S. (2012). ACR/EULAR 2010 rheumatoid arthritis classification criteria. Rheumatology 51, vi5–vi9. 10.1093/rheumatology/kes279 23221588

[B16] Kocijan R. Harre U. Schett G. (2013). ACPA and bone loss in rheumatoid arthritis. Curr. Rheumatol. Rep. 15, 366. 10.1007/s11926-013-0366-7 23955066

[B17] Kume K. Amano K. Yamada S. Kanazawa T. Ohta H. Hatta K. (2014). The effect of tocilizumab on bone mineral density in patients with methotrexate-resistant active rheumatoid arthritis. Rheumatol. Oxf. 53, 900–903. 10.1093/rheumatology/ket468 24441151

[B18] Moshayedi S. Tasorian B. Almasi-Hashiani A. (2022). The prevalence of osteoporosis in rheumatoid arthritis patient: a systematic review and meta-analysis. Sci. Rep. 12, 15844. 10.1038/s41598-022-20016-x 36151246 PMC9508181

[B19] Murakami K. Kobayashi Y. Uehara S. Suzuki T. Koide M. Yamashita T. (2017). A Jak1/2 inhibitor, baricitinib, inhibits osteoclastogenesis by suppressing RANKL expression in osteoblasts *in vitro* . PLoS One 12, e0181126. 10.1371/journal.pone.0181126 28708884 PMC5510865

[B20] Pietschmann P. Butylina M. Kerschan-Schindl K. Sipos W. (2022). Mechanisms of systemic osteoporosis in rheumatoid arthritis. Int. J. Mol. Sci. 23, 8740. 10.3390/ijms23158740 35955873 PMC9368786

[B21] Schett G. McInnes I. B. Neurath M. F. (2021). Reframing immune-mediated inflammatory diseases through signature cytokine hubs. N. Engl. J. Med. 385, 628–639. 10.1056/NEJMra1909094 34379924

[B22] Schulz N. Asendorf T. van Wijnen P. Wilhelmi T. Müller-Ladner U. Lange U. (2025). Bone mineral density during treatment with the janus kinase inhibitor baricitinib in patients with rheumatoid arthritis: a monocentric observational study. Calcif. Tissue Int. 116, 101. 10.1007/s00223-025-01410-9 40694134 PMC12669316

[B23] Smolen J. S. Aletaha D. Bijlsma J. W. Breedveld F. C. Boumpas D. Burmester G. (2010). Treating rheumatoid arthritis to target: recommendations of an international task force. Ann. Rheum. Dis. 69, 631–637. 10.1136/ard.2009.123919 20215140 PMC3015099

[B24] Vasikaran S. Eastell R. Bruyère O. Foldes A. J. Garnero P. Griesmacher A. (2011). Markers of bone turnover for the prediction of fracture risk and monitoring of osteoporosis treatment: a need for international reference standards. Osteoporos. Int. 22, 391–420. 10.1007/s00198-010-1501-1 21184054

[B25] Wettich T. Müller-Ladner U. Dischereit G. Lange U. (2014). Effekte einer Tocilizumab-Therapie auf die Knochendichte, Marker des Knochenstoffwechsels und Aktivitätsparameter bei TNF-α vortherapierter aktiver rheumatoider Arthritis. Aktuelle Rheumatol. 39, 370–374. 10.1055/s-0034-1385942

[B26] Wheater G. Elshahaly M. Naraghi K. Tuck S. P. Datta H. K. van Laar J. M. (2018). Changes in bone density and bone turnover in patients with rheumatoid arthritis treated with rituximab, results from an exploratory, prospective study. PLoS One 13, e0201527. 10.1371/journal.pone.0201527 30080871 PMC6078302

[B27] Wiebe E. Huscher D. Schaumburg D. Palmowski A. Hermann S. Buttgereit T. (2022). Optimising both disease control and glucocorticoid dosing is essential for bone protection in patients with rheumatic disease. Ann. Rheum. Dis. 81, 1313–1322. 10.1136/annrheumdis-2022-222339 35680387 PMC9380479

[B28] Xu M. Tchkonia T. Ding H. Ogrodnik M. Lubbers E. R. Pirtskhalava T. (2015). JAK inhibition alleviates the cellular senescence-associated secretory phenotype and frailty in old age. Proc. Natl. Acad. Sci. U. S. A. 112, E6301–E6310. 10.1073/pnas.1515386112 26578790 PMC4655580

[B29] Zhou P. Zheng T. Zhao B. (2022). Cytokine-mediated immunomodulation of osteoclastogenesis. Bone 164, 116540. 10.1016/j.bone.2022.116540 36031187 PMC10657632

