# Supplementary material for: Skeletal effects of JAK1/2 inhibition versus IL-6 receptor blockade in rheumatoid arthritis: a post hoc comparative cohort analysis based on two prospective observational studies
Source: Front Pharmacol. 2026 May 28;17:1835307. doi: 10.3389/fphar.2026.1835307 (PMC13253618; doi:10.3389/fphar.2026.1835307)
Supplement: Supplementary file 1 [file Table1.docx]

| ***Part A: Lumbar spine BMD*** | | | | | | | | |
| --- | --- | --- | --- | --- | --- | --- | --- | --- |
| **Predictor** | **Primary model** | | | | **Sensitivity model (+MTX)** | | | |
|  | **β** | **95% CI** | **p** | **VIF** | **β** | **95% CI** | **p** | **VIF** |
| Baseline BMD (spine) | −0.080 | [−0.389, +0.229] | 0.603 | 1.15 | −0.071 | [−0.386, +0.244] | 0.648 | 1.17 |
| Treatment (tocilizumab vs. baricitinib) | −0.039 | [−0.459, +0.382] | 0.853 | 2.14 | −0.096 | [−0.587, +0.395] | 0.692 | 2.84 |
| Sex | +0.156 | [−0.170, +0.482] | 0.337 | 1.29 | +0.163 | [−0.169, +0.494] | 0.325 | 1.29 |
| Age | +0.294 | [−0.051, +0.638] | 0.092 | 1.43 | +0.293 | [−0.056, +0.641] | 0.097 | 1.43 |
| Disease duration | −0.158 | [−0.501, +0.185] | 0.355 | 1.42 | −0.172 | [−0.524, +0.180] | 0.328 | 1.46 |
| RF positive | −0.022 | [−0.388, +0.344] | 0.904 | 1.62 | −0.022 | [−0.393, +0.348] | 0.903 | 1.62 |
| ACPA positive | −0.145 | [−0.572, +0.281] | 0.493 | 2.20 | −0.142 | [−0.574, +0.290] | 0.508 | 2.20 |
| ΔDAS28-CRP | −0.359 | [−0.674, −0.044] | **0.027*** | 1.20 | −0.377 | [−0.705, −0.049] | **0.026*** | 1.27 |
| ΔPrednisolone | −0.091 | [−0.475, +0.292] | 0.631 | 1.78 | −0.101 | [−0.492, +0.289] | 0.601 | 1.80 |
|  |  |  |  |  | +0.088 | [−0.285, +0.461] | 0.634 | 1.64 |
| *Model fit: Primary: n=44, R² = 0.320, p(model) = 0.110*  *Sensitivity (+MTX): n=44, R² = 0.324, p(model) = 0.155* | | | | | | | | |

**Supplementary Table S1** Sensitivity analyses including methotrexate use and multicollinearity diagnostics

| ***Part B: Femoral neck BMD*** | | | | | | | | |
| --- | --- | --- | --- | --- | --- | --- | --- | --- |
| **Predictor** | **Primary model** | | | | **Sensitivity model (+MTX)** | | | |
|  | **β** | **95% CI** | **p** | **VIF** | **β** | **95% CI** | **p** | **VIF** |
| Baseline BMD (femoral neck) | −0.129 | [−0.464, +0.207] | 0.441 | 1.23 | −0.136 | [−0.482, +0.210] | 0.431 | 1.27 |
| Treatment (tocilizumab vs. baricitinib) | −0.054 | [−0.528, +0.420] | 0.818 | 2.45 | −0.021 | [−0.588, +0.547] | 0.941 | 3.41 |
| Sex | −0.265 | [−0.588, +0.058] | 0.105 | 1.14 | −0.271 | [−0.605, +0.062] | 0.107 | 1.18 |
| Age | −0.075 | [−0.408, +0.258] | 0.650 | 1.21 | −0.074 | [−0.412, +0.264] | 0.659 | 1.21 |
| Disease duration | −0.019 | [−0.366, +0.328] | 0.912 | 1.31 | −0.014 | [−0.369, +0.340] | 0.936 | 1.33 |
| RF positive | −0.032 | [−0.439, +0.375] | 0.873 | 1.81 | −0.035 | [−0.448, +0.379] | 0.865 | 1.81 |
| ACPA positive | −0.083 | [−0.553, +0.388] | 0.723 | 2.41 | −0.084 | [−0.562, +0.393] | 0.722 | 2.42 |
| ΔDAS28-CRP | −0.204 | [−0.557, +0.150] | 0.250 | 1.36 | −0.196 | [−0.562, +0.170] | 0.284 | 1.42 |
| ΔPrednisolone | +0.258 | [−0.157, +0.674] | 0.215 | 1.88 | +0.269 | [−0.163, +0.701] | 0.215 | 1.98 |
|  |  |  |  |  | −0.043 | [−0.435, +0.348] | 0.823 | 1.62 |
| *Model fit — Primary: n=45, R² = 0.221, p(model) = 0.384*  *Sensitivity (+MTX): n=45, R² = 0.223, p(model) = 0.483* | | | | | | | | |

All values are standardized β-coefficients from multivariable analysis of covariance (ANCOVA) with the 12-month change in bone mineral density (ΔBMD) as the dependent variable. All continuous predictors were standardized prior to analysis (mean = 0, SD = 1). The primary model includes: baseline BMD (site-specific), treatment group, sex, age, disease duration, RF status, ACPA status, ΔDAS28-CRP, and ΔPrednisolone. The sensitivity model additionally includes methotrexate (MTX) use as a binary covariate (0 = no MTX, 1 = MTX). Variance inflation factors (VIF) are reported for each predictor as a measure of multicollinearity; values below 5 indicate acceptable collinearity. Asterisk (*) denotes statistical significance at p < 0.05.
